# Metabolic syndrome and the risk of severe cancer events: a longitudinal study in Japanese workers

**DOI:** 10.1186/s12885-023-11026-7

**Published:** 2023-06-16

**Authors:** Dong Van Hoang, Yosuke Inoue, Ami Fukunaga, Tohru Nakagawa, Toru Honda, Shuichiro Yamamoto, Hiroko Okazaki, Makoto Yamamoto, Toshiaki Miyamoto, Naoki Gommori, Takeshi Kochi, Taiki Shirasaka, Masafumi Eguchi, Takayuki Ogasawara, Kenya Yamamoto, Maki Konishi, Nobumi Katayama, Isamu Kabe, Seitaro Dohi, Tetsuya Mizoue

**Affiliations:** 1 Department of Epidemiology and Prevention, National Center for Global Health and Medicine, Toyama 1-21-1, Shinjuku-ku, Tokyo, 1628655 Japan; 2 Hitachi Health Care Center, Hitachi, Ltd, Ibaraki, Japan; 3 Mitsui Chemicals, Inc, Tokyo, Japan; 4 Yamaha Corporation, Shizuoka, Japan; 5 Nippon Steel Corporation, East Nippon Works, Chiba, Japan; 6 East Japan Works (Keihin), JFE Steel Corporation, Keihin, Kanagawa Japan; 7 Furukawa Electric Co., Ltd, Tokyo, Japan; 8 Mitsubishi Fuso Truck and Bus Corporation, Kawasaki, Kanagawa Japan; 9 Division of Chemical Information, National Institute of Occupational Safety and Health, Kiyose, Kanagawa Japan; 10 Kubota Corporation, Tokyo, Japan

**Keywords:** Metabolic syndrome, Severe cancer events, Mortality, Long-term sick leave, Japan

## Abstract

**Background:**

Metabolic syndrome (MetS) is associated with cancer risk; however, little is known regarding its relationship with the risk of cancer-related premature death and long-term sick leave (LTSL), which can lead to a substantial loss in working years. The present study aimed to quantify the all-site and site-specific associations between MetS and the risk of severe cancer events (a composite outcome of LTSL and mortality due to cancer) in a large working population in Japan.

**Methods:**

We recruited 70,875 workers (59,950 men and 10,925 women), aged 20–59 years, who attended health check-ups in 2011 (10 companies) and 2014 (2 companies). All workers underwent follow up for severe cancer events until March 31, 2020. MetS was defined in accordance with the Joint Interim Statement. Cox regression models were used to quantify the association between baseline MetS and severe cancer events.

**Results:**

During 427,379 person-years of follow-up, 523 participants experienced the outcome consisting of 493 LTSLs of which 124 eventually resulted in death, and 30 deaths without taking LTSL. The adjusted hazard ratios (HR) (95% confidence intervals [CI]) for composite severe events due to all-site, obesity-related, and non-obesity-related cancer among those with vs. without MetS were 1.26 (1.03, 1.55), 1.37 (1.04, 1.82), and 1.15 (0.84, 1.56), respectively. In cancer site-specific analyses, MetS was associated with an increased risk of severe events due to pancreatic cancer (HR, 2.06; 95% CI, 0.99–4.26). When mortality was treated solely as the endpoint, the association was significant for all-site (HR, 1.58; 95% CI, 1.10–2.26), and obesity-related (HR, 1.59; 95% CI, 1.00–2.54) cancer. Additionally, a greater number of MetS components was associated with a greater risk of both severe cancer events and cancer-related mortality (P trend < 0.05).

**Conclusion:**

Among Japanese workers, MetS was associated with an increased risk of severe cancer events, especially those due to obesity-linked cancer.

**Supplementary Information:**

The online version contains supplementary material available at 10.1186/s12885-023-11026-7.

## Background

Metabolic syndrome (MetS), a cluster of cardiometabolic risk factors, [1] is a major public health concern in many countries worldwide [2, 3]. The estimated MetS prevalence among adults in Asia, Europe, and the U.S. are approximately 20%, [2] 24.3%, [4] and 37%,[5] respectively. Epidemiological studies have associated MetS with an increased risk of subsequent chronic conditions (e.g., cardiovascular diseases [6, 7] and osteoporosis 8), and cardiovascular [7,9] and all-cause mortality [7].

Evidence linking MetS to cancer risk has been increasing [10–14]. A meta-analysis of 43 studies [14] reported an association between MetS and an increased incidence of liver cancer (relative risk [RR], 1.43), colorectal cancer (RR, 1.25), bladder cancer (RR, 1.10), and pancreatic cancer (RR, 1.58). A few studies also reported an increase in cancer mortality among people with MetS. [10–13]. Multiple MetS-associated metabolic alterations are involved in the link between MetS and the increased risk of cancer morbidity and mortality [15–17]. For example, a chronic state of low-grade inflammation can cause gene mutations, which in turn can lead to cancer initiation, [18] while excessive synthesis of leptin can facilitate tumor invasion and metastasis [19].

Cancer-related premature death and sick leave are important components of cancer burden in the workplace [20, 21]. For instance, in a Japanese study, death and long-term sick leave (LTSL) due to cancer were the second largest contributors to the total working years lost, accounting for losses of 6.5 (95% CI 5.9–7.3) and 3.2 (95% CI 2.8–3.8) years per 10,000 years, respectively [20]. However, there is little information on the relationship between MetS and such cancer-related events. In our recent study, [22] MetS was associated with increased risk of LTSL due to various causes including cancer; the hazard ratio (HR) for cancer-related LTSL was 1.24 (95% CI 1.00–1.53). Although both cancer-related LTSL and premature death are severe events resulting from cancer progression, the latter confers a much greater burden in the workplace [20]. Therefore, investigations into the effect of MetS on the outcome of cancer progression should consider both cancer-related LTSL and premature cancer mortality. It is also important to identify the specific types of cancer that can be attributed to MetS.

In the present study, we aimed to extend the current understanding of the relationship between MetS and cancer by quantifying the all-site and site-specific association between MetS and the risk of severe cancer events (i.e., a composite outcome of LTSL and premature mortality due to cancer) in a large working population in Japan.

## Methods

### Study setting

The present prospective cohort analysis was part of the Japan Epidemiology Collaboration on Occupational Health (J-ECOH) Study, an ongoing company-based research project investigating health determinants among workers in Japan. The study population comprised workers in various industrial sectors (e.g., electric machinery and apparatus manufacturing; steel, chemical, gas, and nonferrous metal manufacturing; automobile and instrument manufacturing; plastic product manufacturing; and healthcare) [23–25]. The follow-up data at each participating company were collected through annual health check-ups consisting of anthropometric measurements, physical examinations, laboratory tests, and a self-administered questionnaire regarding medical history and health-related lifestyle.

Prior to data collection at each participating company, we displayed posters explaining the purpose and procedure of the J-ECOH study. We informed workers that their health-related data (e.g., health check-ups, sick leaves, or mortality) owned by the company would be anonymized and provided to the J-ECOH Study and that they should notify their occupational physicians of their disagreement, if any. This opt-out procedure complied with the Japanese Ethical Guidelines for Epidemiological Research for observational studies that use existing data. The study protocol was approved by the Ethics Committee of the National Center for Global Health and Medicine, Japan (approval number: NCGM-G-001140).

### Analytic cohort

In April 2012, we created a within-study registry to collect information on mortality and sick leave events (date and medically certified causes) reported by the participating companies. We used the latest health check-up information before the launch of the disease registry to define the cohort, i.e., health check-up information collected in 2011 for individuals working for 10 companies where the registry was launched in April 2012 and health check-up information in 2014 for those working for two other companies where the registry was launched in April 2015. We defined eligible participants as those who were under the management, in relation to their health checkups, of the occupational physicians at each participating company.

**Fig. 1:**
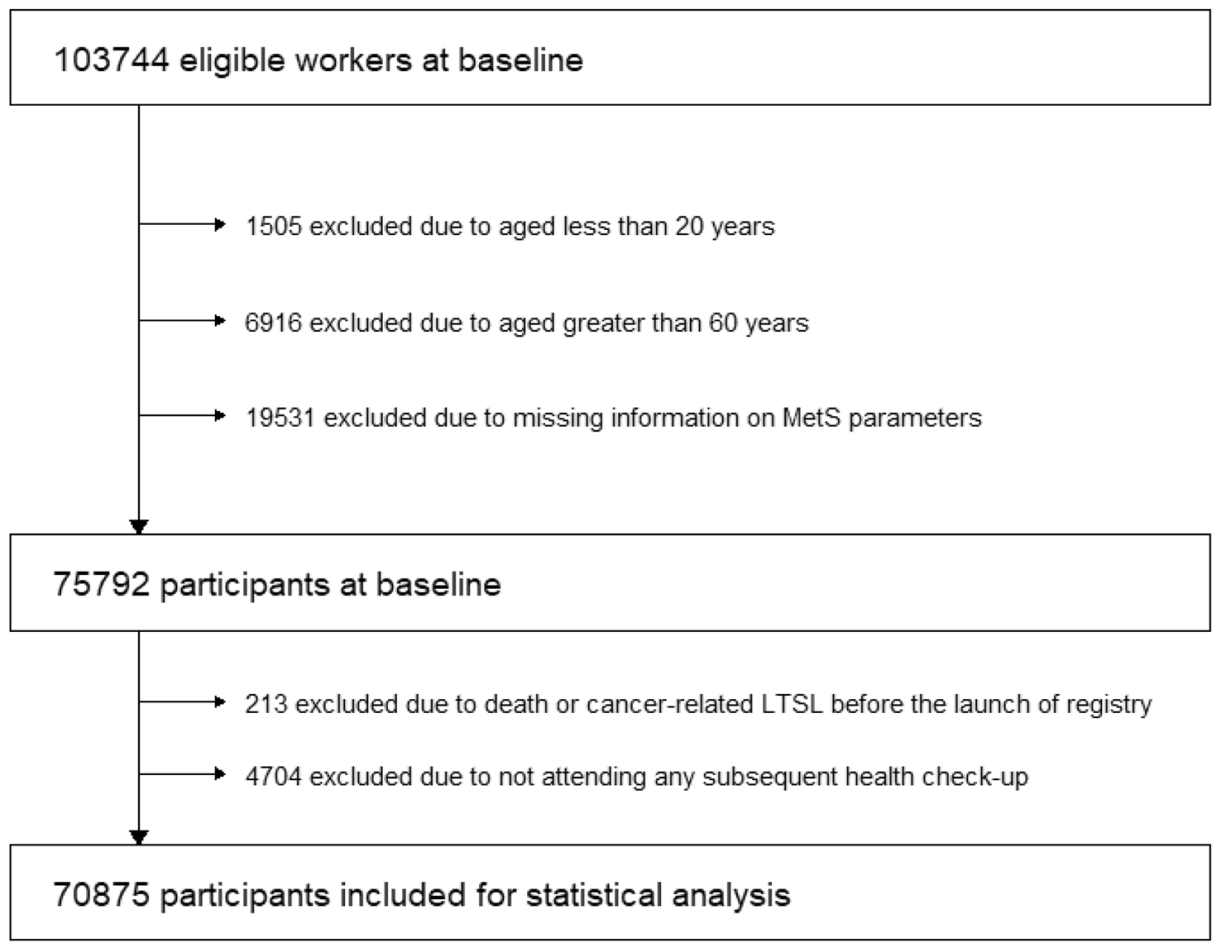
Participants in the Japan Epidemiology Collaboration on Occupational Health Study (n = 70,875), Kanto and Tokai, Japan, FY2012-2020. MetS, metabolic syndrome; FY, Japanese fiscal year, from April 1 to March 31 of the following year. The baseline health check-up was conducted in FY2011 (10 companies) and FY2014 (2 companies), and the follow-up started from April 1, 2012 (11 companies) and April 1, 2015 (two companies) to March 31, 2020

At baseline, 103,744 workers (86,748 men and 16,996 women) were eligible for the present study. Among them, we excluded those aged < 20 years (i.e., legal smoking age; n = 1,505), those aged ≥ 60 years (who were about to retire and tended not to take LTSL; n = 6,916), those with missing information on MetS components (n = 19,531). We further excluded those who died or experienced LTSL before the launch of registry (n = 213) or those who did not attend any subsequent health check-ups (n = 4704), leaving 70,875 participants (59,950 men and 10,925 women) for statistical analysis (Fig. 1). Those who were excluded tended to be younger and never smoker, and had a lower prevalence of cancer, cardiovascular diseases, and psychiatric disorders (Table S1).

### General health examination

Body weight and height were measured in light clothing and without shoes, respectively. Body mass index (BMI) was calculated as weight in kilograms divided by height in meters squared. Waist circumference (WC) was measured at the umbilical level using a measuring tape with the participants in the standing position. Blood pressure (BP) was measured by a trained nurse in a sitting position using either an automatic or mercury sphygmomanometer. Fasting plasma glucose (FPG) levels were measured using either the enzymatic or glucose oxidase peroxidative electrode method. Triglycerides (TG) and high-density lipoprotein cholesterol (HDL) levels were measured using an enzymatic method.

### Metabolic syndrome and covariates

MetS was defined in accordance with the Joint Interim Statement, [26] as a clustering of any three or more of the following components: high FPG (≥ 100 mg/dL or use of antidiabetic medication), central obesity (WC ≥ 90 cm for men or ≥ 80 cm for women), high TG (≥ 150 mg/dL or using lipid-lowering medication), high BP (systolic BP ≥ 130 mmHg, diastolic BP ≥ 85 mmHg or using antihypertensive medication), and low HDL (< 40 mg/dL for men or < 50 mg/dL for women). The cutoff values for WC were based on the recommendations of the World Health Organization for Asian populations [27].

Information on covariates was collected during annual health check-ups organized by each company. While the selection of specific questions was not under our control, we harmonized some variables to ensure that responses were comparable across the study site. Based on epidemiological evidence for their association with cancer events and their availability from the participating companies, age, sex, [28] smoking, [29] and pre-existing cancer [30] were selected as covariates since their information was available from all companies. Smoking status (never, former, or current) was identified from the self-administered questionnaires at baseline which was consistently applied across participating companies.

### Ascertainment of cancer mortality and LTSL

LTSL has no universal definition, but it has been commonly defined as a sick leave of at least 4 weeks [31]. Thus, for this study, we defined LTSL as a sick leave of ≥ 30 consecutive days, a threshold at which all the participating companies were able to provide information. The causes of cancer mortality and LTSL were classified according to the International Classification of Diseases, 10th Revision (ICD-10), i.e., C00-C99, D00-D09 (causes of LTSL) and D37-D48 [32]. Cancer events were further categorized into obesity-related cancers (cancers of the mouth [C04], pharynx [C14], esophagus [C15], stomach [C16], colon and rectum [C18, C20], liver [C22, including intrahepatic bile duct, C22.1], gallbladder [C23], pancreas [C25], larynx [C32], breast cancer [C50], endometrium [C54], ovary [C56], prostate [C61], and kidney [C64]), [33] other cancers, and lung cancer (C34).

In the present study, the primary composite endpoint was the occurrence of all-site severe cancer events (either the first LTSL or mortality due to cancer, whichever occurred first), and the secondary composite endpoint was the occurrence of site-specific severe cancer events (i.e., events due to obesity-related cancer or cancer of the colorectum, stomach, pancreas, liver, breast, or lung).

### Statistical analysis

The background characteristics of the study population, according to MetS status, were described as the mean and standard deviation (SD) for continuous variables and as percentages for categorical variables. Person-time was calculated from April 1, 2012 (10 companies) and April 1, 2015 (2 companies) to the date of severe cancer events (date of death or the starting date of the first LTSL whichever came first) or the date of censoring, which was either the last annual health check-up, death due to other causes, or the end of follow-up (March 31, 2020; three companies ended the follow-up earlier). When premature cancer mortality was treated as a single endpoint, the person-time was calculated from the starting date of follow up to the date of death, regardless of LTSL status.

Cox proportional hazard regression was used to estimate the HRs and 95% CIs for all-site and site-specific severe cancer events, comparing participants with and without MetS. The proportional hazard assumption for MetS was tested using Schoenfeld residuals; and we confirmed that the assumption was not violated (P value = 0.89). To account for missing data on smoking status (n = 1,345), we used multiple imputation to create 20 datasets [34] and combined them according to Rubin’s rule [35]. The main analyses consisted of two models: Model 1 was adjusted for age, sex, and worksite (worksites with less than 10 cancer events were grouped together), and Model 2 was further adjusted for smoking status and pre-existing cancer. For each cancer site-specific analysis (e.g., events due to obesity-related cancer), those who had events due to other cancers were censored on the date of the event. As a secondary analysis, we investigated the all-site and site-specific associations between MetS and cancer mortality (treated as a single endpoint). The population attributable fraction (PAF) was calculated using the following formula: PAF = pd x (HR − 1/HR), where pd is the proportion of cases exposed to the risk factor, and HR is the adjusted HR in Model 2 [36].

We also examined the associations between the number of MetS components and all-site severe cancer events and between the number of MetS components and cancer mortality, while combining those with at least four components into one group. Trends in these associations were assessed by assigning an ordinal number (1–5) to each group, which was treated as a continuous variable when fitting the regression models.

The association between MetS and cancer events in men might be different from that in women. However, nearly 85% of the present analytical sample was men, and thus we conducted a sensitivity analysis in men only, instead of stratifying by sex. To eliminate the potential effect of pre-existing cancer or severe health conditions on the association between MetS and severe cancer events, we conducted a series of sensitivity analyses using Model 1 and Model 2, in which we first excluded participants with known cancer at baseline, then we excluded those who died within the first year of follow up. Statistical significance was set at P < 0.05 (two-tailed). All statistical analyses were conducted in RStudio (version 3.2.4) using the package “survival” (version 3.1.8). [37].

## Results

The baseline characteristics of the study participants, stratified by MetS status, are presented in Table 1. Participants who had MetS were more likely to be older, men, overweight or obese, and former or current smokers, and they had a higher prevalence of baseline cancer, cardiovascular diseases, or psychiatric disorders than those without MetS.

**Table 1:** Baseline characteristics of study participants

Characteristics	All participants	MetS status		
		Yes	No	
N	70,875	12,059	58,816	
Age, mean [SD]	44.3 (9.0)	48.0 (7.5)	43.6 (9.1)	
Sex (men)	59,950 (84.6)	11,137 (92.4)	48,813 (83.0)	
Body mass index (kg/m^2^)				
< 18.5	3271 (4.6)	25 (0.2)	3246 (5.5)	
18.5–24.9	47,589 (67.1)	3146 (26.1)	44,443 (75.6)	
25.0–29.9	16,712 (23.6)	6567 (54.5)	10,145 (17.2)	
≥ 30.0	3303 (4.7)	2321 (19.2)	982 (1.7)	
Smoking status				
Never-smoker	29,632 (41.8)	3819 (31.7)	25,813 (43.9)	
Former smoker	17,599 (24.8)	3520 (29.2)	14,079 (23.9)	
Current smoker	23,644 (33.4)	4720 (39.1)	18,924 (32.2)	
History of cancer	671 (0.9)	147 (1.2)	524 (0.9)	
History of cardiovascular disease	734 (1.0)	274 (2.3)	460 (0.8)	
History of psychiatric disorder	1079 (1.5)	246 (2.0)	833 (1.4)	
MetS components				
High BP ^a^	23,580 (33.3)	9584 (79.5)	13,996 (23.8)	
High FPG ^b^	23,737 (33.5)	9290 (77.0)	14,447 (24.6)	
High TG ^c^	19,749 (27.9)	9556 (79.2)	10,193 (17.3)	
Central obesity ^d^	16,715 (23.6)	8818 (73.1)	7897 (13.4)	
Low HDL ^e^	5693 (8.0)	3425 (28.4)	2268 (3.9)	
Number of MetS components				
0	24,064 (34.0)		24,064 (40.9)	
1	20,703 (29.2)		20,703 (35.2)	
2	14,049 (19.8)		14,049 (23.9)	
3	8143 (11.5)	8143 (67.5)		
4	3336 (4.7)	3336 (27.7)		
5	580 (0.8)	580 (4.8)		

Figures are n (%), unless otherwise stated; MetS: Metabolic syndrome; ^a^ systolic blood pressure ≥ 130 mmHg or diastolic blood pressure ≥ 85 mmHg or using antihypertensive medication; ^b^ fasting plasma glucose ≥ 100 mg/dL or using antidiabetic medication; ^c^ triglyceride ≥ 150 mg/dL or using lipid lowering medication; ^d^ waist circumference ≥ 90 cm (for men) or ≥ 80 cm (for women); ^e^ high-density lipoprotein cholesterol < 40 mg/dL (for men) or < 50 mg/dL (for women)

During 427,379 person-years of follow-up, 523 participants experienced severe cancer events (493 LTSL and 154 deaths). Of those who died from cancer, 124 (80.5%) had undergone LTSL due to cancer (Table S2). The overall incidence rates for composite cancer events and mortality were 1.22 (95% CI: 1.12–1.33), 0.36 (95% CI: 0.30–0.42) per 1,000 person-years, respectively. The specific cancer diagnoses are shown in Supplementary Table S3.

Table 2 presents the associations between MetS and the risk of all-site and site-specific severe cancer events. Participants with MetS exhibited a significantly higher risk of severe events due to all-site cancer (HR 1.26; 95% CI, 1.03–1.55) and obesity-related cancer (HR 1.37; 95% CI, 1.04–1.82) than those without MetS, although there were no differences in the risk of non-obesity-related cancer between the groups (HR 1.15; 95% CI, 0.84–1.56). In cancer site-specific analyses, MetS seemed to be associated with an increased risk of severe events due to the cancer at all common sites (i.e., colorectum [HR 1.37], stomach [HR 1.42], liver [HR 1.26], breast [HR 1.33], and lung [HR 1.19]), but the associations were not statistically significant, except for that concerning pancreatic cancer (HR 2.06; 95% CI, 0.99–4.26). Similarly, MetS was associated with a significantly increased risk of mortality due to all-site cancer (HR 1.58; 95% CI, 1.10–2.26) and obesity-related cancer (HR, 1.59; 95% CI, 1.00–2.54), but not with an increased risk of non-obesity-related cancer (HR 1.56; 95% CI, 0.89–2.74). MetS was also associated with an increased risk of pancreatic cancer mortality, but this association was not statistically significant (HR, 2.18; 95% CI, 0.87–5.44). Additionally, the MetS-attributed PAFs for composite severe events due to all-site, obesity-related, and pancreatic cancers were 7.9%, 10.9%, and 26.8%, respectively. The MetS-attributed PAFs for mortality due to all-site and obesity-related cancer were 16.1% and 16.4%, respectively.

**Table 2:** Association between MetS and cancer events in Japanese workers (N = 70,875)

Cancer	HR (95% CI)					
	Mortality/LTSL			Mortality		
	MetS (-)	MetS (+)		MetS (-)	MetS (+)	
No. of participants	58,816	12,059		58,816	12,059	
Person-years	357,065	70,314		363,988	72,407	
**Overall cancer**						
No. of events	400	123		110	44	
Model 1	1.00 (ref)	1.28 (1.04, 1.57)		1.00 (ref)	1.57 (1.10, 2.25)	
Model 2	1.00 (ref)	1.26 (1.03, 1.55)		1.00 (ref)	1.58 (1.10, 2.26)	
PAF (%)		**7.9**			**16.1**	
**Obese-related cancer** ^**a**^						
No. of events	208	68		68	26	
Model 1	1.00 (ref)	1.38 (1.04, 1.83)		1.00 (ref)	1.57 (0.99, 2.49)	
Model 2	1.00 (ref)	1.37 (1.04, 1.82)		1.00 (ref)	1.59 (1.00, 2.54)	
PAF (%)		**10.9**			**16.4**	
*Colorectal cancer*						
No. of events	54	18		14	5	
Model 1	1.00 (ref)	1.34 (0.78, 2.32)		1.00 (ref)	1.50 (0.53, 4.29)	
Model 2	1.00 (ref)	1.37 (0.80, 2.37)		1.00 (ref)	1.63 (0.57, 4.68)	
PAF (%)		-			-	
*Gastric cancer*						
No. of events	40	16		14	7	
Model 1	1.00 (ref)	1.46 (0.81, 2.64)		1.00 (ref)	1.96 (0.77, 4.94)	
Model 2	1.00 (ref)	1.42 (0.79, 2.56)		1.00 (ref)	1.94 (0.77, 4.92)	
PAF (%)		-			-	
*Pancreatic cancer*						
No. of events	21	12		12	8	
Model 1	1.00 (ref)	2.06 (1.00, 4.27)		1.00 (ref)	2.18 (0.88, 5.44)	
Model 2	1.00 (ref)	2.06 (0.99, 4.26)		1.00 (ref)	2.18 (0.87, 5.44)	
PAF (%)		**26.8**			-	
*Liver cancer*						
No. of events	16	6		10	4	
Model 1	1.00 (ref)	1.27 (0.49, 3.29)		1.00 (ref)	1.47 (0.45, 4.78)	
Model 2	1.00 (ref)	1.26 (0.48, 3.26)		1.00 (ref)	1.49 (0.45, 4.85)	
PAF (%)		-			-	
*Breast cancer* ^**b**^						
No. of events	26	4		7	1	
Model 1	1.00 (ref)	1.59 (0.54, 4.72)		1.00 (ref)	1.42 (0.16, 12.34)	
Model 2	1.00 (ref)	1.33 (0.44, 3.98)		1.00 (ref)	0.50 (0.06, 4.13)	
PAF (%)		-			-	
**Non-obesity-related cancer**						
No. of events	192	55		42	18	
Model 1	1.00 (ref)	1.17 (0.86, 1.59)		1.00 (ref)	1.58 (0.90, 2.78)	
Model 2	1.00 (ref)	1.15 (0.84, 1.56)		1.00 (ref)	1.56 (0.89, 2.74)	
PAF (%)		-			-	
*Lung cancer*						
No. of events	53	18		19	8	
Model 1	1.00 (ref)	1.23 (0.71, 2.12)		1.00 (ref)	1.54 (0.66, 3.57)	
Model 2	1.00 (ref)	1.19 (0.69, 2.05)		1.00 (ref)	1.46 (0.63, 3.40)	
PAF (%)		-			-	

MetS: metabolic syndrome; LTSL: long-term sick leave; Model 1: adjusted for age, sex and worksite; Model 2: further adjusted for smoking status and pre-existing cancer; PAF: population attributable fraction;

^**a**^ included the cancer of mouth, pharynx, oesophagus, stomach, colon and rectum, liver, gallbladder, pancreas, larynx, breast cancer, endometrium, ovary, prostate and kidney; ^**b**^ in women only

The sensitivity analyses in men only resulted in similar pattern of association between MetS and cancer events (Table S4), e.g., the adjusted HRs (95% CI) for severe cancer events due to all-site, obesity-related, and pancreatic cancer were 1.28 (1.03–1.60), 1.40 (1.03–1.89), and 1.94 (0.91–4.13), respectively. In the sensitivity analyses excluding participants with pre-existing cancer (Table S5) or those who died within the first year of follow-up (Table S6), significant associations became more apparent. For example, the adjusted HRs (95% CIs) for severe cancer events, and mortality due to pancreatic cancer were 2.31 (95% CI, 1.10–4.84), and 2.63 (1.02–6.81), respectively (Table S5).

**Fig. 2:**
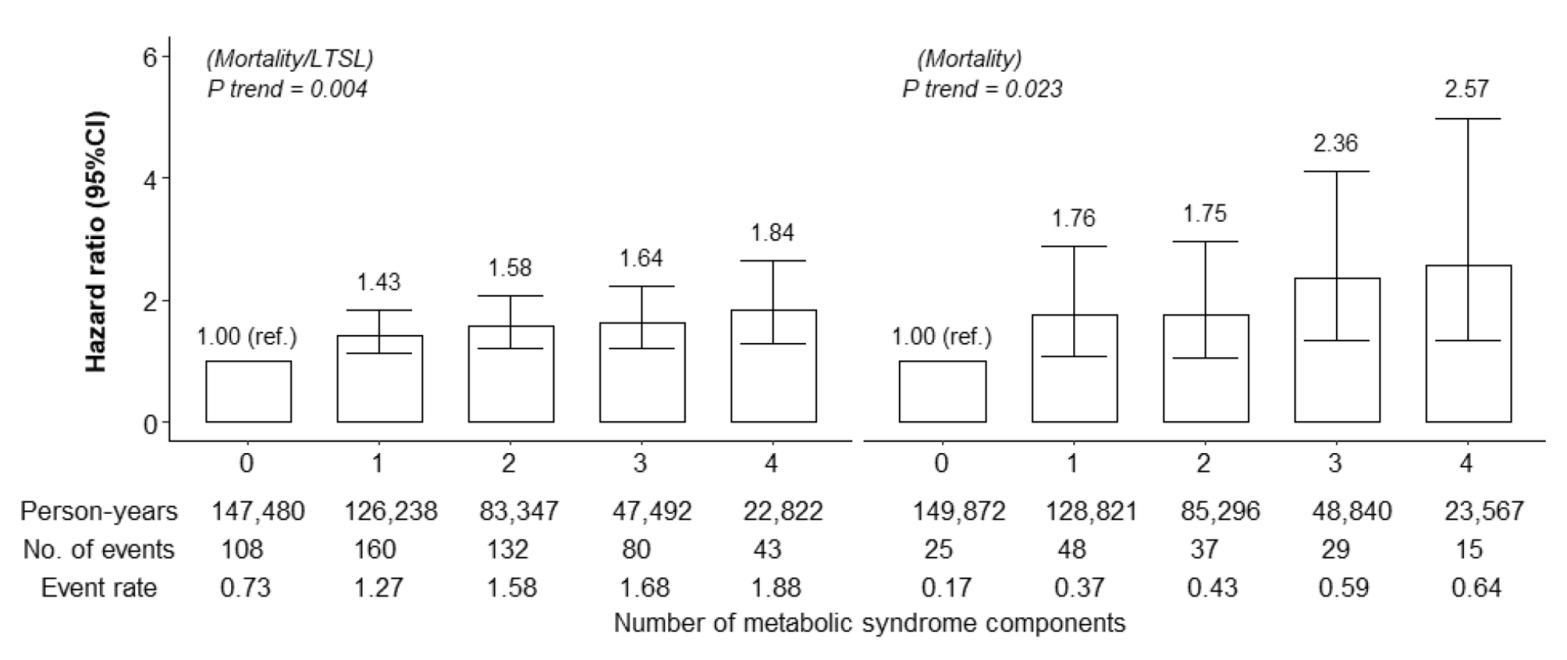
Multivariable-adjusted hazard ratios for the cancer events associated with the number of metabolic syndrome components among Japanese workers (n = 70,875, Japan, FY2012-2020). Cancer events: composite outcome of mortality and/or long-term sick leave. The Cox regression model was adjusted for age, sex, smoking status, and pre-existing cancer; event rate: per 1,000 person-years; and metabolic syndrome components: high blood pressure (systolic blood pressure ≥ 130 mmHg or diastolic blood pressure ≥ 85 mmHg or use of antihypertensive medication), high fasting glucose (≥ 100 mg/dL or using antidiabetic medication), central obesity (waist circumference ≥ 90 cm for men or ≥ 80 cm for women), high triglyceride (≥ 150 mg/dL or using lipid-lowering medication), and reduced high-density lipoprotein cholesterol (< 40 mg/dL for men or < 50 mg/dL for women)

As shown in Fig. 2, there were significant associations between the number of MetS components and the risk of severe cancer events, and between the number of MetS components and the risk of premature cancer mortality. For example, compared with those without MetS components, the adjusted HRs for severe cancer events among those with one, two, three, and four or five components were 1.43, 1.58, 1.64, and 1.84, respectively (P_trend_ = 0.004).

## Discussion

In the present large-scale prospective cohort study conducted among Japanese workers, MetS was associated with a significantly increased risk of composite severe events of LTSL and deaths due to cancer, especially events due to obesity-related cancers. Additionally, the number of MetS components was significantly associated with an increase in the number of composite cancer events. When mortality was treated as a single endpoint, MetS was associated with a significantly higher risk of all-site and obesity-related cancer mortality. To the best of our knowledge, this study is the first to investigate the relationship between MetS and composite severe cancer events.

The present association between MetS and composite cancer events agrees with epidemiological data linking MetS to morbidity, [14] progression, [38–41] and mortality [10, 13, 42, 43]. For example, studies have reported that MetS is significantly associated with an increased risk of cancer at various sites (e.g., the liver, colorectum, pancreas, breast, and prostate gland), [14] the progression of prostate cancer (HR 2.77), [44] and the mortality of all-site cancer (HR 1.33) [10]. The present finding was further supported by our observation of a dose–response association between the number of MetS components and the increased risk of severe cancer events that had not been investigated in previous studies.

Obesity increases the risk of various malignancies (i.e., the cancer of the mouth, pharynx, esophagus, stomach, colon and rectum, liver, gallbladder, pancreas, larynx, breast cancer, endometrium, ovary, prostate and kidney) [33]. The association between MetS and composite events due to obesity-related cancers observed in the present study is compatible with the epidemiological data linking MetS to obesity-related malignancies [11,12, 14, 45]. For example, a meta-analysis of 43 studies [14] showed an association between MetS and the morbidity of liver (RR 1.43), bladder (RR 1.10), pancreatic (RR 1.58), colorectal (RR 1.25), endometrial (RR 1.61), and breast (RR 1.56) cancer. A U.S. study [45] also reported the association of MetS with mortality for obesity-related cancer (HR 1.65). The null association observed in the present study between MetS and the events of non-obesity-related malignancies, among which lung cancer was the most frequent (28.8%), is in line with those of previous studies showing no association between MetS and the morbidity [14, 46] or mortality [10] of lung cancer. This similarity indicates that MetS may increase cancer risk through the mechanism by which obesity promotes carcinogenesis.

The observed association between MetS and cancer mortality is also in accordance with those reported in previous prospective studies [10, 13, 42, 43]. In the U.S., the RR (95% CI) for cancer mortality associated with MetS was 1.33 (1.11–1.59) [10]. In Japan, two population-based studies (mean age of 53.4 and 57.5 years) [12, 47] reported inconsistent data: In one study, MetS was associated with cancer mortality in women (HR 1.69; 95% CI, 1.21–2.36) but not in men (HR 1.21; 95% CI, 0.90–1.62) [12]; in the other, no associations were observed (HR 1.06; 95% CI, 0.82–1.36) [47]. It should be noted that those Japanese studies defined MetS based on BMI. As the present study utilized WC (a standard indicator of abdominal obesity and a better predictor of cancer risk than BMI 48), its results provide more reliable evidence regarding the association between MetS and cancer mortality in the Japanese working population.

The association with MetS appears to be stronger for cancer mortality (HR 1.58) than for composite cancer events (HR 1.26), which largely reflects the risk associated with LTSL. This finding indicates that MetS may be associated with deadly forms of cancer. In fact, some studies have shown a link between MetS and worse oncologic outcomes, such as aggressive tumor features of prostate cancer [39] or advanced stages of bladder cancer [49]. Given the limited evidence on this issue, more studies are required to confirm or refute whether MetS is more strongly associated with aggressive forms of cancer than with milder ones.

The association between MetS and an increased risk of severe cancer events may be explained by several potential biological mechanisms [15, 16]. MetS is associated with chronic inflammation, which can promote the damage of deoxyribonucleic acid leading to cancer initiation [17, 18]. MetS, especially in the presence of central obesity, can lead to increased levels of circulating insulin-like growth factor I [50] which can in turn contribute to the invasion, angiogenesis, and metastasis of cancer [51]. Studies have also suggested that the overproduction of leptin, a hormone-like cytokine synthesized by adipocytes, in the context of MetS may be another important promotor for the invasion and metastasis of malignant tumours [19]. Such multistage involvement of metabolic abnormalities in the carcinogenic process may jointly explain the higher risk of severe forms of cancer among persons with MetS.

LTSL and death due to cancer significantly contribute to the total loss of working years [20,21]. If the association observed in the present study is causal, the prevention of MetS may help mitigate the burden of cancer in the workplace. In 2008, the Specific Health Checkups and Specific Health Guidance program targeting MetS was implemented in Japan [52]. Studies conducted since its inception have demonstrated that the implementation of this program has been associated with improvements in metabolic components such as WC [53, 54] and HDL-C. With the present findings, such programs are also expected to mitigate the burden of severe cancer events in the workplace, although further studies are required to confirm this hypothesis.

The present study has several strengths. Our analyses were based on a well-defined cohort of more than 100,000 employees. Information on cancer events was extracted from company records, and the cause of LTSL was determined according to medical certificates. The participating companies have a well-organized management system for LTSL that enables us to precisely capture cancer events. Nonetheless, our study had some limitations. First, we adjusted for a limited number of covariates (age, sex, smoking status, and pre-existing cancer), and thus cannot exclude the possibility of unmeasured confounding factors, such as history of cancer-related LTSL, socioeconomic status, and occupation. Second, due to concerns about stigma, attending physicians may have avoided documenting cancer as an underlying condition on medical certificates. Third, we did not assess the change in the status of MetS during the follow-up course, which might have affected the risk of cancer events. Finally, most study participants were male workers (85%), meaning that the results should be generalized with caution.

## Conclusions

The current results suggest that MetS is associated with an increased risk of severe cancer events, especially events due to obesity-linked malignancies, among Japanese workers. The risk of severe cancer events may also increase with an increase in the number of MetS components.

## Electronic supplementary material

Below is the link to the electronic supplementary material.

Supplementary tables

## Data Availability

The datasets analyzed during the current study are not publicly available due the ethical requirements of the research proposal but are available from the National Center for Global Health and Medicine on reasonable request.

## Abbreviations

BMI: Body mass index
BP: Blood pressure
FPG: Fasting plasma glucose
HDL: High-density lipoprotein
ICD-10: International Classification of Diseases, 10th Revision
J-ECOH: Japan Epidemiology Collaboration on Occupational Health
LTSL: Long-term sick leave
MetS: Metabolic syndrome
PAF: Population attributable fraction
RR: Relative risk
SD: Standard deviation
TG: Triglycerides
WC: Waist circumference
