# Supplementary material for: Metabolic syndrome and the risk of severe cancer events: a longitudinal study in Japanese workers
Source: BMC Cancer. 2023 Jun 16;23:555. doi: 10.1186/s12885-023-11026-7 (PMC10273588; doi:10.1186/s12885-023-11026-7)
Supplement: Supplementary file 1 — Supplementary tables [file 12885_2023_11026_MOESM1_ESM.docx]

# Supplementary tables

## Table S1: Baseline characteristics of eligible participants according to exclusion status

| **Characteristics** | **Included participants** | **Excluded participants** |
| --- | --- | --- |
| N | 70875 | 32869 |
| Age (year), mean [SD] | 44.3 (9.0) | 38.5 (15.3) |
| Sex (men) | 59950 (84.6) | 26798 (81.5) |
| Body mass index (kg/m^2^) |  |  |
| < 18.5 | 3271 (4.6) | 2060 (6.3) |
| 18.5 - 24.9 | 47589 (67.1) | 22729 (69.2) |
| 25.0 - 29.9 | 16712 (23.6) | 6535 (19.9) |
| ≥ 30.0 | 3303 (4.7) | 1381 (4.2) |
| Missing |  | 164 (0.5) |
| Smoking status |  |  |
| Never smoker | 29086 (41.0) | 14714 (44.8) |
| Former smoker | 17308 (24.4) | 6296 (19.2) |
| Current smoker | 23136 (32.6) | 9005 (27.4) |
| Missing | 1345 (1.9) | 2854 (8.7) |
| History of cancer | 671 (0.9) | 307 (0.9) |
| History of cardiovascular disease | 734 (1.0) | 297 (0.9) |
| History of psychiatric disorders | 1079 (1.5) | 317 (1.0) |
| Metabolic syndrome status |  |  |
| No | 58816 (83.0) | 9862 (30.0) |
| Yes | 12059 (17.0) | 2875 (8.7) |
| Missing information |  | 20,132 |
|  | | |

## Table S2. Cancer deaths by LTSL status

| **Mortality status** | **LTSL status** | | | **Total** |
| --- | --- | --- | --- | --- |
|  | Non-events | Cancer events | Non-cancer events |  |
| Non-events | 67650 | 369 | 2518 | **70537** |
| Cancer events | 30 | 124 | 0 | **154** |
| Non-cancer event | 147 | 0 | 37 | **184** |
| **Total** | **67827** | **493** | **2555** | **70875** |

## Table S3. Site-specific cancer causes of deaths and long-term sick leave

| **Cancer site** | **ICD-10** | **Number of events** | |  |
| --- | --- | --- | --- | --- |
|  |  | LTSL/mortality | LTSL | Mortality |
| Colorectum | C18, C20 | 72 | 67 | 19 |
| Bronchus and lung | C34 | 71 | 66 | 27 |
| Stomach | C16 | 56 | 50 | 21 |
| Pancreas | C25 | 33 | 27 | 20 |
| Breast | C50 | 30 | 29 | 8 |
| Liver | C22 | 22 | 20 | 14 |
| Oesophagus | C15 | 19 | 18 | 6 |
| Hematopoietic and lymphoid tissues | C85 | 18 | 18 | 0 |
| Brain and central nervous system | D43 | 15 | 15 | 3 |
| Prostate gland | C61 | 15 | 14 | 1 |
| Plasma cells | C90 | 10 | 10 | 2 |
| Female genital organs | D39 | 9 | 9 | 0 |
| Myeloid blood cells | C92 | 9 | 9 | 3 |
| Oral cavity and digestive organs | D37 | 9 | 9 | 0 |
| Ovary | C56 | 9 | 9 | 2 |
| Biliary tract | C24 | 8 | 8 | 6 |
| Bladder | C67 | 8 | 8 | 1 |
| Kidney | C64 | 7 | 6 | 2 |
| Corpus uteri | C54 | 6 | 6 | 0 |
| Other connective and soft tissue | C49 | 5 | 5 | 0 |
| Secondary malignancy | C78 | 5 | 5 | 1 |
| Others |  | 87 | 85 | 18 |
| **Total** |  | **523** | **493** | **154** |
| ICD-10: International classification of diseases; LTSL: long-term sick leave | | | |  |

| Table S4: Association between MetS and cancer events in men (N = 59,950) | | | | |
| --- | --- | --- | --- | --- |
|  | **Hazard ratio (95% confidence interval)** | | | |
|  | LTSL/Mortality | | Mortality | |
|  | MetS(-) | MetS(+) | MetS(-) | MetS(+) |
| No. of participants | 48,813 | 11,137 | 48,813 | 11,137 |
| Person-years | 299,791 | 65,154 | 305,355 | 67,102 |
| **Overall cancer** |  |  |  |  |
| No. of events | 306 | 111 | 95 | 42 |
| Model 1 | 1.00 (ref) | 1.29 (1.04, 1.61) | 1.00 (ref) | 1.62 (1.12, 2.35) |
| Model 2 | 1.00 (ref) | 1.28 (1.03, 1.60) | 1.00 (ref) | 1.63 (1.13, 2.37) |
| **Obese-related cancers ^a^** |  |  |  |  |
| No. of events | 152 | 60 | 54 | 25 |
| Model 1 | 1.00 (ref) | 1.40 (1.03, 1.89) | 1.00 (ref) | 1.71 (1.06, 2.78) |
| Model 2 | 1.00 (ref) | 1.40 (1.03, 1.89) | 1.00 (ref) | 1.75 (1.08, 2.85) |
| *Colorectal cancer* |  |  |  |  |
| No. of events | 46 | 17 | 12 | 5 |
| Model 1 | 1.00 (ref) | 1.41 (0.80, 2.48) | 1.00 (ref) | 1.75 (0.60, 5.12) |
| Model 2 | 1.00 (ref) | 1.44 (0.82, 2.55) | 1.00 (ref) | 1.94 (0.66, 5.69) |
| *Gastric cancer* |  |  |  |  |
| No. of events | 36 | 15 | 12 | 7 |
| Model 1 | 1.00 (ref) | 1.44 (0.78, 2.65) | 1.00 (ref) | 2.23 (0.86, 5.75) |
| Model 2 | 1.00 (ref) | 1.41 (0.76, 2.59) | 1.00 (ref) | 2.24 (0.86, 5.81) |
| *Pancreatic cancer* |  |  |  |  |
| No. of events | 19 | 11 | 11 | 8 |
| Model 1 | 1.00 (ref) | 1.93 (0.91, 4.12) | 1.00 (ref) | 2.30 (0.91, 5.80) |
| Model 2 | 1.00 (ref) | 1.94 (0.91, 4.13) | 1.00 (ref) | 2.29 (0.91, 5.81) |
| *Liver cancer* |  |  |  |  |
| No. of events | 16 | 6 | 10 | 4 |
| Model 1 | 1.00 (ref) | 1.27 (0.49, 3.29) | 1.00 (ref) | 1.47 (0.45, 4.78) |
| Model 2 | 1.00 (ref) | 1.26 (0.48, 3.26) | 1.00 (ref) | 1.49 (0.45, 4.85) |
| **Non-obese cancers** |  |  |  |  |
| No. of events | 154 | 51 | 41 | 17 |
| Model 1 | 1.00 (ref) | 1.19 (0.86, 1.64) | 1.00 (ref) | 1.50 (0.84, 2.67) |
| Model 2 | 1.00 (ref) | 1.17 (0.85, 1.61) | 1.00 (ref) | 1.48 (0.83, 2.64) |
| *Lung cancer* |  |  |  |  |
| No. of events | 47 | 17 | 19 | 7 |
| Model 1 | 1.00 (ref) | 1.23 (0.70, 2.15) | 1.00 (ref) | 1.31 (0.54, 3.17) |
| Model 2 | 1.00 (ref) | 1.18 (0.67, 2.08) | 1.00 (ref) | 1.25 (0.52, 3.03) |

MetS: metabolic syndrome; LTSL: long-term sick leave; Model 1: adjusted for age and worksite; Model 2: further adjusted for smoking status and pre-existing cancer; **^a^** included the cancer of mouth, pharynx, oesophagus, stomach, colon and rectum, liver, gallbladder, pancreas, larynx, endometrium, ovary, prostate, and kidney.

| Table S5: Association between MetS and cancer events, excluding baseline cancer (N = 70,204) | | | | | |
| --- | --- | --- | --- | --- | --- |
|  | **Hazard ratio (95% confidence interval)** | | | | |
|  | **LTSL/Mortality** | | **Mortality** | | |
|  | **MetS(-)** | **MetS(+)** | **MetS(-)** | **MetS(+)** | |
| No. of participants | 58,292 | 11,912 | 58,292 | 11,912 | |
| Person-years | 354,230 | 69,565 | 361,067 | 71,601 | |
| **Overall cancer** |  |  |  |  | |
| No. of events | 377 | 115 | 98 | 40 | |
| Model 1 | 1.00 (ref) | 1.28 (1.04, 1.59) | 1.00 (ref) | 1.63 (1.12, 2.37) | |
| Model 2 | 1.00 (ref) | 1.26 (1.02, 1.57) | 1.00 (ref) | 1.62 (1.11, 2.36) | |
| **Obese-related cancers ^a^** |  |  |  |  | |
| No. of events | 198 | 64 | 60 | 24 | |
| Model 1 | 1.00 (ref) | 1.36 (1.02, 1.82) | 1.00 (ref) | 1.63 (1.01, 2.66) | |
| Model 2 | 1.00 (ref) | 1.35 (1.01, 1.80) | 1.00 (ref) | 1.64 (1.01, 2.67) | |
| *Colorectal cancer* |  |  |  |  | |
| No. of events | 53 | 16 | 13 | 4 | |
| Model 1 | 1.00 (ref) | 1.23 (0.69, 2.17) | 1.00 (ref) | 1.38 (0.44, 4.35) | |
| Model 2 | 1.00 (ref) | 1.25 (0.71, 2.22) | 1.00 (ref) | 1.49 (0.47, 4.70) | |
| *Gastric cancer* |  |  |  |  | |
| No. of events | 39 | 16 | 13 | 7 | |
| Model 1 | 1.00 (ref) | 1.49 (0.82, 2.69) | 1.00 (ref) | 2.09 (0.81, 5.34) | |
| Model 2 | 1.00 (ref) | 1.45 (0.80, 2.62) | 1.00 (ref) | 2.06 (0.80, 5.27) | |
| *Pancreatic cancer* |  |  |  |  | |
| No. of events | 19 | 12 | 10 | 8 | |
| Model 1 | 1.00 (ref) | 2.32 (1.11, 4.87) | 1.00 (ref) | 2.66 (1.03, 6.88) | |
| Model 2 | 1.00 (ref) | 2.31 (1.10, 4.84) | 1.00 (ref) | 2.63 (1.02, 6.81) | |
| *Liver cancer* |  |  |  |  | |
| No. of events | 16 | 6 | 10 | 4 | |
| Model 1 | 1.00 (ref) | 1.27 (0.49, 3.28) | 1.00 (ref) | 1.46 (0.45, 4.77) | |
| Model 2 | 1.00 (ref) | 1.26 (0.48, 3.26) | 1.00 (ref) | 1.49 (0.45, 4.85) | |
| *Breast cancer* |  |  |  |  | |
| No. of events | 22 | 3 | 4 | 0 | |
| Model 1 | 1.00 (ref) | 1.49 (0.43, 5.15) | 1.00 (ref) | 0.00 (0.00, Inf) | |
| Model 2 | 1.00 (ref) | 1.50 (0.43, 5.19) | 1.00 (ref) | 0.00 (0.00, Inf) | |
| **Non-obese cancers** |  |  |  |  |  |
| No. of events | 179 | 51 | 38 | 16 |  |
| Model 1 | 1.00 (ref) | 1.20 (0.87, 1.65) | 1.00 (ref) | 1.62 (0.89, 2.93) |  |
| Model 2 | 1.00 (ref) | 1.17 (0.85, 1.61) | 1.00 (ref) | 1.58 (0.87, 2.87) |  |
| *Lung cancer* |  |  |  |  |  |
| No. of events | 48 | 17 | 17 | 7 |  |
| Model 1 | 1.00 (ref) | 1.34 (0.76, 2.35) | 1.00 (ref) | 1.56 (0.64, 3.84) |  |
| Model 2 | 1.00 (ref) | 1.28 (0.73, 2.25) | 1.00 (ref) | 1.48 (0.60, 3.63) |  |
| MetS: metabolic syndrome; LTSL: long-term sick leave; Model 1: adjusted for age, sex and worksite; Model 2: further adjusted for smoking status; **^a^** included the cancer of mouth, pharynx, oesophagus, stomach, colon and rectum, liver, gallbladder, pancreas, larynx, endometrium, ovary, prostate, and kidney. | | | | |  |

## Table S6: Association between MetS and cancer events in Japanese workers, excluding baseline pre-existing cancer or death within the first year of follow-up (N =70,109)

| **Cancer site** | **Hazard ratio (95% confidence interval)** | | | | |  |
| --- | --- | --- | --- | --- | --- | --- |
|  | **Cancer M-LTSL** | |  | **Cancer mortality** | | |
|  | MetS (-) | MetS (+) |  | MetS (-) | MetS (+) | |
| No. of participants | 58,227 | 11,882 |  | 58,227 | 11,882 | |
| Person-years | 354,206 | 69,554 |  | 361,029 | 71,584 | |
| **Overall cancer** |  |  |  |  |  | |
| No. of events | 364 | 111 |  | 88 | 36 | |
| Model 1 | 1.00 (ref) | 1.29 (1.04, 1.60) |  | 1.00 (ref) | 1.65 (1.11, 2.45) | |
| Model 2 | 1.00 (ref) | 1.27 (1.02, 1.58) |  | 1.00 (ref) | 1.63 (1.10, 2.43) | |
| **Obese-related cancer** |  |  |  |  |  | |
| No. of events | 186 | 62 |  | 51 | 22 | |
| Model 1 | 1.00 (ref) | 1.42 (1.06, 1.91) |  | 1.00 (ref) | 1.83 (1.09, 3.06) | |
| Model 2 | 1.00 (ref) | 1.41 (1.05, 1.89) |  | 1.00 (ref) | 1.83 (1.10, 3.07) | |
| *Colorectum* |  |  |  |  |  | |
| No. of events | 49 | 16 |  | 10 | 4 | |
| Model 1 | 1.00 (ref) | 1.35 (0.76, 2.40) |  | 1.00 (ref) | 2.01 (0.60, 6.66) | |
| Model 2 | 1.00 (ref) | 1.37 (0.77, 2.44) |  | 1.00 (ref) | 2.13 (0.64, 7.09) | |
| *Stomach* |  |  |  |  |  | |
| No. of events | 35 | 15 |  | 11 | 6 | |
| Model 1 | 1.00 (ref) | 1.55 (0.84, 2.87) |  | 1.00 (ref) | 2.16 (0.78, 5.96) | |
| Model 2 | 1.00 (ref) | 1.50 (0.81, 2.78) |  | 1.00 (ref) | 2.10 (0.76, 5.83) | |
| *Pancreas* |  |  |  |  |  | |
| No. of events | 17 | 12 |  | 8 | 8 | |
| Model 1 | 1.00 (ref) | 2.63 (1.23, 5.62) |  | 1.00 (ref) | 3.36 (1.23, 9.14) | |
| Model 2 | 1.00 (ref) | 2.64 (1.23, 5.63) |  | 1.00 (ref) | 3.36 (1.23, 9.16) | |
| *Liver* |  |  |  |  |  | |
| No. of events | 15 | 5 |  | 9 | 3 | |
| Model 1 | 1.00 (ref) | 1.12 (0.40, 3.12) |  | 1.00 (ref) | 1.21 (0.32, 4.59) | |
| Model 2 | 1.00 (ref) | 1.11 (0.40, 3.10) |  | 1.00 (ref) | 1.24 (0.33, 4.71) | |
| PAF (%) |  |  |  |  |  | |
| *Breast cancer* | 22 | 3 |  | 4 | 0 | |
| No. of events | 1.00 (ref) | 1.49 (0.43, 5.16) |  | 1.00 (ref) | 0.00 (0.00, Inf) | |
| Model 1 | 1.00 (ref) | 1.50 (0.43, 5.19) |  | 1.00 (ref) | 0.00 (0.00, Inf) | |
| Model 2 |  |  |  |  |  | |
| **Non-obesity-related cancer** | 178 | 49 |  | 37 | 14 | |
| No. of events | 1.00 (ref) | 1.15 (0.83, 1.59) |  | 1.00 (ref) | 1.42 (0.76, 2.65) | |
| Model 1 | 1.00 (ref) | 1.13 (0.82, 1.56) |  | 1.00 (ref) | 1.38 (0.74, 2.59) | |
| Model 2 |  |  |  |  |  | |
| *Lung* | 47 | 17 |  | 16 | 7 | |
| No. of events | 1.00 (ref) | 1.36 (0.77, 2.39) |  | 1.00 (ref) | 1.63 (0.66, 4.02) | |
| Model 1 | 1.00 (ref) | 1.30 (0.74, 2.29) |  | 1.00 (ref) | 1.54 (0.63, 3.81) | |
| Model 2 | 364 | 111 |  | 88 | 36 | |
| MetS: metabolic syndrome; LTSL: long-term sick leave; Model 1: adjusted for age, sex and worksite; Model 2: further adjusted for smoking status; **^a^** included the cancer of mouth, pharynx, oesophagus, stomach, colon and rectum, liver, gallbladder, pancreas, larynx, breast cancer, endometrium, ovary, prostate and kidney; **^b^** in women only | | | | | |  |
